# Microbial cell-free DNA-sequencing as an addition to conventional diagnostics in neonatal sepsis

**DOI:** 10.1038/s41390-024-03448-1

**Published:** 2024-08-14

**Authors:** Julian Balks, Silke Grumaz, Sonia Mazzitelli, Ulrike Neder, Lotte Lemloh, Tamene Melaku, Kirsten Glaser, Andreas Mueller, Florian Kipfmueller

**Affiliations:** 1 Division of Neonatology and Pediatric Intensive Care, Children’s Hospital, University of Bonn, Bonn, Germany; 2 Institute of Medical Microbiology, Immunology and Parasitology (IMMIP), University Hospital Bonn, Bonn, Germany; 3 Noscendo, Duisburg, Germany; 4 Division of Neonatology, Department of Women’s and Children’s Health, University Medical Center Leipzig, Leipzig, Germany

## Abstract

**Background:**

Bloodstream infections remain a challenge for neonatologists, as traditional culture-based methods are time-consuming and rely on adequate blood volume. Next-generation sequencing (NGS) offers an alternative, as it can identify microbial cell-free DNA (mcfDNA) in a small blood sample, providing rapid pathogen detection. This study aimed to assess the diagnostic performance of DISQVER®-NGS compared to blood cultures in neonatal patients with suspected sepsis.

**Methods:**

In neonates with suspected sepsis, blood cultures and samples for NGS were prospectively collected. Patients were divided into four categories: 1) sepsis, blood culture positive, 2) clinical sepsis, culture negative, 3) suspected sepsis, 4) validation cohort.

**Results:**

NGS detected bacterial, viral or fungal mcfDNA in 24 of 82 samples. Blood cultures were collected in 46 of 84 patients (15/46 positive). DISQVER® correctly identified pathogens in 9/15 patients with a positive blood culture, two with intrinsic resistance to their antibiotic regimen. In seven samples NGS reported the mcfDNA of bacteria that could have theoretically grown in culture but did not.

**Conclusions:**

NGS may enhance sensitivity in sepsis diagnostics by detecting mcfDNA in neonates with suspected sepsis. Interpreting NGS results requires correlation with clinical data, laboratory values, and routine microbiological tests for a comprehensive understanding of the patient’s condition.

**Impact:**

- Conventional blood culture methods have limitations in accuracy and turnaround time.
- The study aimed to investigate the diagnostic performance of the Next-Generation Sequencing method DISQVER® compared to traditional blood cultures in neonatal patients with suspected sepsis.
- Our findings suggest that NGS has the potential to augment the precision of conventional diagnostic techniques, can lead to improved detection of pathogens and targeted treatment approaches in neonatal sepsis.
- It is emphasized that further validation and integration with clinical and microbiological data are required to ensure optimal clinical utility.

## Introduction

Bloodstream infections (BSIs) remain a major challenge for term and especially preterm infants in neonatal intensive care units. Hospitalized neonates and very immature preterm infants are at highest risk.^1,2^ Despite the availability of a variety of antibiotics and empiric antibiotic regimens for different foci of infection, neonatal sepsis is still a major cause of neonatal morbidity and mortality worldwide.^3–7^ The clinical presentation of neonatal sepsis is nonspecific and variable.^7^ Timely initiation of appropriate antimicrobial treatment is critical to prevent acute and long-term organ dysfunction as well as life-threatening deterioration. Studies demonstrated that delayed initiation of empiric antibiotic treatment for suspected neonatal sepsis was associated with increased sepsis-related mortality.^8^ While prompt empiric antibiotic administration improves patient survival, the chance of culturing the causative pathogen of sepsis decreases after initiation, warranting the collection of blood cultures before antibiotics are administered.^9^ Also, treatment with broad-spectrum empiric antibiotics must be balanced against potential side-effects including antibiotic selection pressure, the development of multi-drug resistant bacteria, antibiotic associated toxicities or negative impacts on the microbiome.^7,10,11^ Ultimately, the identified pathogen should guide the selection of an individual antibiotic regime.

Blood cultures remain the “gold standard” for the detection of bacteremia. Cultural diagnostic techniques offer several advantages, such as being relatively cost-effective compared to nucleic acid amplification methods or DNA sequencing and allow testing of antibiotic susceptibilities. However, the fundamental problem with blood cultures is that growth is dependent on time and blood culture volume.^12,13^ Despite advances in culturing techniques bacterial growth may not occur due to preanalytical errors, lack of certain growth requirements, the use of antibiotics prior to sample collection or, in particular in neonates, insufficient blood culture volume.^12^

Sequencing-based diagnostic approaches can identify pathogens from peripheral blood and may offer an alternative to conventional blood cultures. The comparatively small volume of blood required for pathogen-detection by sequencing of microbial cell-free DNA (mcfDNA) is particularly important in premature and newborn infants.^14,15^ Sequencing of cell-free DNA (cfDNA) and bioinformatic filtering of raw reads is followed by qualitative assessment of the mcfDNA, potentially enabling pathogen identification and quantification. Assessing the read count of individual pathogens is one way to distinguish between infection and contamination.^16^ Sequencing of mcfDNA from patients’ blood samples ^17^ is a promising approach with limited published data in neonates.^18,19^ Because the method sequences mcfDNA fragments rather than live pathogens, it can provide accurate results even after antimicrobial therapy has been initiated.^20^ Next-Generation Sequencing (NGS) could shorten the turnaround time of modern sepsis diagnostics, allowing for faster de-escalation to pathogen-directed therapy^19^ or termination of antibiotic treatment.

Aim of this prospective feasibility study was to investigate the diagnostic performance of the NGS method DISQVER® compared to blood cultures in neonatal patients with suspected sepsis.

## Methods

### Study design and patients

The study was designed as a prospective, single-center study. Neonates admitted during the first 28 days of life to the neonatal intensive care unit (level III) of the University Hospital Bonn (UKB) receiving a diagnostic workup for suspected late-onset sepsis (LOS) at an age of 72 h to 3 months were included in the study. Between September 2020 and July 2022, 125 patients were enrolled in the study. The ethics committee of the University of Bonn approved the study (Study registration number: 114/22). Written informed consent was obtained from parents or legal representatives before participation in the study.

In this study, sepsis was defined according to the established criteria of the German neonatal infection surveillance system (NEO-KISS).^21,22^ These criteria include clinical signs, blood culture findings, laboratory markers (CRP, IL-6, leukocyte and thrombocyte count) in line with other sepsis classifications.^6,23^ Patients were allocated to one of three groups: 1) blood culture-positive sepsis, 2) blood culture-negative sepsis, 3) suspected sepsis. Additionally, healthy newborns without infection were recruited as a validation group to establish initially required sample preparation quality parameters and baselines for microbial cell-free DNA. These patients were allocated to group 4): patients without clinical signs of infection (Table 1).

**Table 1:** Patient classification.

Group	Description	Definition
1	Sepsis, blood culture positive	Episodes in which patients had at least one positive blood culture and clinical signs of sepsis or necrotizing enterocolitis.
2	Sepsis, blood culture negative	Episodes in which patients had negative blood cultures but had clinical signs of sepsis or necrotizing enterocolitis and were treated with antibiotics for at least 5 days.
3	Suspicion of Sepsis	Episodes in which patients were suspected to have a sepsis, were started on antibiotic therapy, but whose therapy lasted less than 5 days because the treating physicians reassessed when infection was considered unlikely.
4	Validation group	Patients without clinical signs of sepsis

Of the 125 enrolled patients, 57 blood cultures and 134 samples for mcfDNA sequencing were collected. Six patients were included twice in the study due to a second episode of suspected sepsis, while one patient was included three times for the same reason. Overall, eight samples were added from repeatedly tested patients.

**Fig. 1:**
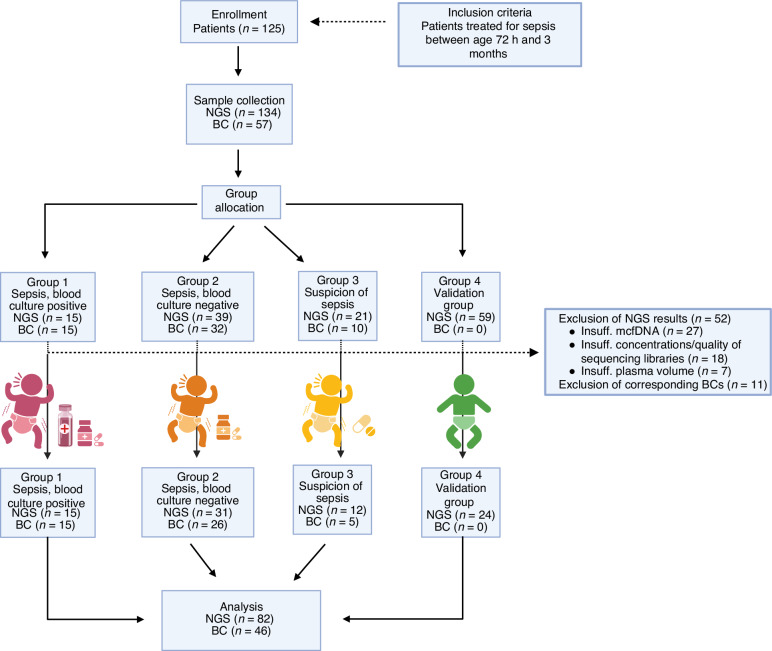
Study flowchart. Between September 2020 and July 2022, 125 patients were enrolled and allocated to 4 groups. Sampling yielded 57 blood culture and 134 mcfDNA samples. Due to secondary septic episodes 6 patients were included twice and one patient three times. Overall, 8 samples were added from repeatedly tested patients. Due to insufficient plasma volume (*n* = 7), insufficient cell-free DNA (*n* = 27), and insufficient quality of sequencing libraries (*n* = 18) 52 NGS samples and their corresponding blood culture results (where available) were excluded (*n* = 11). Leaving 82 NGS and 46 BC results for comparative analysis. Abbreviations: NGS Next-Generation sequencing, BC blood culture, insuff. insufficient, n number of samples.

Exclusion criteria: Infants older than 3 months or patients receiving palliative care were excluded from the study. Other reasons for exclusion were an inability to perform NGS due to insufficient plasma volume (*n* = 7), insufficient amounts of cell-free DNA (*n* = 27) or insufficient concentrations/quality of sequencing libraries (*n* = 18). Among these 52 NGS samples which were not considered for further analysis, 35 belonged to the validation group (group 4), while 8 and 9 were from group 2 and group 3, respectively. Along with the 52 NGS samples also their matching blood cultures (where available) were excluded (*n* = 11, 6 from group 2 and 5 from group 3), this included 1 duplicate sample (Fig. 1).

Finally, mcfDNA sequencing was performed in 82 blood samples from 74 (41 male and 33 female) patients (Table 2 and Supplementary Table S2). Out of the 82 mcfDNA sequencing results, 46 could be matched to blood culture results. This was due to several reasons such as the absence of blood cultures in the validation group, occasional unsuccessful attempts to collect blood cultures, inability to collect blood cultures due to the patient’s clinical condition, or inadequate clinical suspicion of an infection to warrant blood culture collection. Due to the aforementioned reasons five patients from group 2 and seven patients from group 3 did not have a blood culture drawn during their infectious episode.

**Table 2:** Overview group characteristics

	Overall	Group 1	Group 2	Group 3	Group 4
Patients	74	14	25	12	23
NGS samples analyzed	82	15	31	12	24
NGS positive	24	11	8	3	2
BC samples analyzed	46	15	26	5	0
BC positive	15	15	0	0	0
Gestational age (median [IQR] in weeks)	35.1[27.0–39.0]	35.0 [26.9–37.9]	32.1 [24.4–36.0]	33.5 [29.6–34.9]	39.56 [38.9–40.0]
Age at sample collection (median [IQR] in days)	6 [3–42]	34 [11–61]	10 [3–62]	5 [3–36]	3 [3]
Birth weight (median [IQR] in kg)	2.52 [0.95–3.52]	1.27 [0.79–3.06]	1.37 [0.75–3.28]	1.85 [1.26–2.44]	3.65 [3.45–3.85]
Survival	82.9% (68/82)	60.0% (9/15)	77.4% (24/31)	91.7% (11/12)	100% (24/24)

Overall, 74 patients yielded 82 NGS and 46 BC results. Due to a second episode of suspected sepsis, six patients were included in the study twice and one patient three times, adding eight samples from repeatedly tested patients and explaining the discrepancy between numbers of patients and samples per group (74 patients and 82 samples). *NGS* Next-Generation sequencing, *cfDNA* cellfree DNA, *BC* blood culture, *IQR* interquartile range.

As many NGS results, including the validation collective, could not be matched to BCs due to a lack of BC results, a case-by-case assessment was performed additionally, serving as a composite reference standard. To evaluate the plausibility of the DISQVER® test, results obtained by sequencing were compared to 1) blood culture results, 2) results of other routine microbiological or virologic examinations and 3) the patient’s clinical course, the combination of all 3 together with the physicians’ assessment serving as a composite reference standard.

### Routine sepsis diagnostics and blood cultures

When sepsis was clinically suspected, blood samples were routinely collected for sepsis evaluation. Inflammatory markers (CRP, IL-6), a complete blood count (CBC) and blood cultures were obtained (BD BACTEC™ Peds Plus™—inoculation volume 0.5 ml for preterm and 1.0 ml for term neonates). Blood samples for the validation group were taken from healthy newborns at the same time of the newborn bloodspot screening (for congenital metabolic disorders) and did not include the collection of a blood culture. Blood samples were sent to the central laboratory at University Hospital Bonn for measurement of inflammatory markers (CRP, IL-6, CBC) while the blood cultures were sent to the Institute of Medical Microbiology, Immunology and Parasitology (IMMIP) at Bonn University Hospital immediately after collection. Blood cultures were incubated in the BD Bactec FX (Becton, Dickinson and Company) according to standard protocol for a maximum of 5 days (120 h). Identification of organisms was achieved by MALDI-TOF MS (matrix-assisted laser desorption/ionization time of flight mass spectrometry—bioMérieux VITEK® MS). The VITEK® 2 XL (bioMérieux) was used for automated susceptibility testing. Antibiotic regimens administered were documented for all cases. In instances where antibiotic treatment occurred before sampling, the potential impact on the detection rates of NGS and blood cultures was evaluated. Antibiotic susceptibility testing results were documented for patients with positive blood cultures to assess the efficacy of empiric antibiotic treatments provided. To distinguish between true infection and contamination, several factors were evaluated: growth of the same organism in another microbiologic sample collected within 2 weeks prior or after collection of NGS and BC (e.g., another blood culture, tracheal secretion, tip of removed central veinous catheter, screening swabs), the patients’ clinical condition or the presence of implanted devices and the possibility of catheter related blood stream infections (CRBSI) and time to positivity of blood cultures (growth in <96 h).

For confirmation or exclusion of suspected viral etiologies, samples such as tracheal secretions, anal swabs, cerebrospinal fluid or EDTA plasma were sent to the Virology department at Bonn University Hospital for PCR diagnostics.

### Next-generation sequencing diagnostics

The blood samples required for NGS of microbial cell-free DNA were collected simultaneously with and at the same site as the routine blood samples for sepsis examination. In our study, we collected 0.5 ml blood in Sarstedt K3 EDTA sample tubes (Sarstedt, Nümbrecht, Germany). Collected blood samples for NGS were centrifuged at 3000 rpm for 10 min. Plasma was frozen at −80 °C and later transported to the collaborating laboratory of Noscendo in Tuebingen, Germany, for sequencing and bioinformatic evaluation with their DISQVER® platform optimized for neonatal samples. For sequencing, the frozen plasma samples were thawed on ice, centrifuged at 16.000 rpm for 10 min at 4 °C to remove remaining cellular debris and/or cryoprecipitates and supernatants were transferred to fresh reaction tubes. 100 µl was brought to 1100 µl with sterile 1 x PBS (Thermo Fisher Scientific, Waltham, Massachusetts) and DNA was isolated with the QIAsymphony DSP Circulating DNA kit (Qiagen, Hilden, Germany) on a QIAsymphony DSP instrument (Qiagen, Hilden, Germany). DNA concentrations were measured using a Qubit dsDNA HS assay (Thermo Fisher Scientific, Waltham, Massachusetts), and samples having a concentration higher or equal to 0.03 ng/µl were used for library preparation on a Hamilton NGS STAR (Hamilton, Bonaduz, Switzerland) instrument. Sequencing libraries were again quantified and quality assessed using the aforementioned Qubit kit as well as the Fragment Analyzer HS NGS Fragment assay (Agilent, Santa Clara, California) on a Fragment Analyzer 5300 instrument (Agilent, Santa Clara, California). Subsequently, samples that passed our quality assessment were sequenced with 75 bp read length and a minimum of 25 Mio reads per sample on an Illumina NextSeq550 instrument (Illumina, San Diego, California).

Raw sequencing data was subjected to various QC controls comprising, PHRED score filtering, adapter trimming, complexity filtering as well as k-mer-based contamination screening. To pass the quality filter, read quality needs to surpass a Phred score of 20 and achieve a minimal length of 50 bp after quality control. All data generated was analyzed using Noscendo’s CE-IVD platform DISQVER®.

All DISQVER® results were compared to the corresponding BC results. We evaluated whether the results were concordant (same pathogen detected by blood culture and NGS analysis) or discordant (pathogen only detected by one technique or different pathogens detected). In all cases, the plausibility of the BC and NGS results were evaluated based on the patient’s clinical course, underlying disease, and viral or microbiological diagnostic tests performed 2 weeks prior to or after BC and NGS sample collection (anal swabs, nasopharyngeal swabs, tracheal secretions, etc.).

## Results

The median gestational age in our cohort was 35.1 weeks (IQR 27.0–39.0), with 25 of 74 patients born earlier than 30 weeks gestation (Table 2 and Supplementary Table S2). Median age at sample acquisition was 6 days (IQR 3–42). Apart from prematurity, the most common underlying conditions were of gastrointestinal origin (including necrotizing enterocolitis, intestinal or colonic perforation, meconium ileus, esophageal atresia, Hirschsprungs’ disease), followed by malformations or conditions of the heart and lung (congenital diaphragmatic hernia, hypoplastic left heart syndrome, Tetralogy of Fallot, persistent ductus arteriosus, ventricular septal defect, congenital chylothorax, pneumothorax, pneumonia—Supplementary Table S2).

### Results of blood cultures

Blood cultures were obtained in 46 of 82 cases, 15 turned positive (32.6%) while 31 remained negative (67.4%). One blood culture grew more than one bacterium. 14 of 15 blood cultures revealed the growth of only one pathogen. In the corresponding plasma samples, DISQVER® detected mcfDNA of the same bacterium in 5 of 15 cases, while mcfDNA of several bacteria (including the bacteria grown in culture) was detected in 4 of 15 samples (Table 3). In 6 cases, there was either a negative DISQVER® result (4/6 false negative) or the DISQVER® and blood culture result differed (2/6). Two blood cultures taken from a patient with a 14-day interval revealed *S. epidermidis*, DISQVER® results were negative in both instances. Two other cultures grew *E. coli* and *K. pneumoniae*, respectively but had a negative DISQVER® result. When *A. ursingii* grew in culture, DISQVER® reported reads of Cytomegalovirus (CMV). In the one culture that exhibited growth of multiple bacteria (*E. cloacae complex, S. epidermidis* and *S. captitis*), sequencing revealed cfDNA of *Corynebacterium otitidis, Alloiococcus otitidis, Raoultella planticola* and *Corynebacterium afermentans*. All blood culture results were deemed true positive. The growth of *S. epidermidis* in two cultures was deemed positive due to a short time to positivity (17 h and 37 h respectively) and matching clinical picture of a catheter-associated infection. Where possible, discrepant samples were repeated for NGS analysis. With exception of the two cases of *S. epidermidis* positive blood culture, where not enough remnant material was left, the other four samples were repeated and initial DISQVER® results were confirmed.

**Table 3:** Comparison of positive blood culture with DISQVER® results (Group 1).

Sample number	Antibiotic prior to sampling	Pathogen in blood culture (BC)	Plausibility	Resistance to empiric antibiotics after sampling	Detection of microbial cfDNA in NGS	Evaluation of NGS results	Concordance between BC and NGS
2	No	*Klebsiella oxytoca*	Yes	No Van + Pip/Taz	*Klebsiella oxytoca, Klebsiella pneumoniae, Staphylococcus epidermidis*	true positive	Yes
3^a^	No	*Staphylococcus epidermidis*	Yes	No Van + Pip/Taz	Negative	false negative	No
4^a^	Yes Van + Pip/Taz	*Staphylococcus epidermidis*^b^	Yes	No Van + Mero	Negative	false negative	No
16	No	*Enterobacter cloacae complex*	Yes	No Van + Pip/Taz	*Enterobacter cloacae, Staphylococcus hominis, Enterobacter hormaechei, Staphylococcus epidermidis, Streptococcus salivarius, Enterococcus faecalis, Streptococcus mitis, Klebsiella pneumoniae*	true positive	Yes
57	No	*Candida albicans*	Yes	Yes^c^ Van + Mero	*Candida albicans*	true positive	Yes
89	No	*Enterobacter cloacae complex, Staphylococcus capitis, Staphylococcus epidermidis*	Yes	No Van + Pip/Taz	*Corynebacterium otitidis, Alloiococcus otitis, Raoultella planticola, Corynebacterium afermentans*	differing results	No
104	Yes Van + Pip/Taz	*Klebsiella pneumonia*^b, d^	Yes	No Van + Pip/Taz + Fluco	*Klebsiella pneumoniae*^d^	true positive	Yes
106	No	*Staphylococcus epidermidis*	Yes	No Van + Mero	*Stapyhlococcus epidermidis, Bifidobacterium longum, Klebsiella pneumonia, Streptcoccus gwangjuense, Serratia marcescens, Staphylococcus haemolyticus, Corynebacterium striatum, Enterococcus faecalis, Gardnerella vaginalis*	true positive	Yes
107	No	*Enterobacter cloacae complex*	Yes	No Van + Mero	*Enterobacter spp*.	true positive	Yes
112	No	*Serratia marcescens*	Yes	No Van + Pip/Taz	*Serratia marcescens, Staphylococcus haemolyticus, Staphylococcus epidermidis, Enterococcus faecium*	true positive	Yes
114	No	*Klebsiella pneumoniae*	Yes	No Van + Pip/Taz	Negative	false negative	No
116	Yes Amp/Sul + Tobra	*Serratia marcescens*^d, e^	Yes	Yes Amp/Sul + Tobra	*Serratia marcescens*^d^	true positive	yes
121	Yes Van + Pip/Taz	*Acinetobacter ursingii*^e^	Yes	Yes Van + Pip/Taz	*Human Cytomegalovirus*	differing results	No
125	No	*Escherichia coli*	Yes	No Van + Pip/Taz	Negative	false negative	No
134	Yes Amp/Sul	*Escherichia coli*^d^	Yes	Yes Amp/Sul	*Escherichia coli*^d^	true positive	Yes
	**5/15**	**bacteria 14/15** **fungi 1/15**	**plausible 15/15**	**Resistance 4/15**	**NGS positive 11/15** **NGS negative 4/15**	**true positive 9/15** **false negative 4/15** **differing 2/15**	**concordant 9/15**

Concordant results of NGS and BC were found in 9 out of 15 samples (NGS detected cfDNA of the same pathogen that was found in blood culture). The NGS results of these nine cases were deemed true positive. In 6 out of 15 cases, DISQVER® showed false negative results (4/6) or results differed from blood culture (2/6). In five cases antibiotics were administered prior to sampling. Plausibility of blood culture results was assessed by infectious disease experts and microbiologists, based on underlying disease, patient’s clinical course and routine microbiologic tests performed within two weeks of sample collection. *Enterobacter cloacae* complex: Seven species of the genus *Enterobacter* belong to the *Enterobacter cloacae complex (ECLCO)*: *E. cloacae, E. asburiae, E. hormaechei, E. kobei, E. ludwigii, E. nimipressuralis, E. mori*. These species share phenotypic and, importantly, genotypic characteristics. MALDI-TOF MS often identifies *ECLCO* but may fail to reliably identify the different species that comprise the complex. Molecular methods like PCR or sequencing identify the genetic differences within the complex and can thus more reliably determine which species of ECLCO is present. For this reason, *Enterobacter cloacae* detected in blood culture is often reported as *ECLCO*, while NGS often reports the same bacteria to the exact species of *ECLCO*. This difference is of no clinical or therapeutic concern.

*Van* Vancomycin, *Pip/Taz* Piperacillin/Tazobactam, *Amp/Sul* Ampicillin/Sulbactam, *Tobra* Tobramycin, *Mero* Meropenem, *Fluco* Fluconazol, *NGS* Next-Generation sequencing, *BC* blood culture.

^a^Sample numbers 3 and 4 were collected from the same patient during two different septic episodes 14 days apart.

^b^Bacteria grew in 2 of 5 pre-treated cultures despite the bacterias’ susceptibility to the therapy.

^c^A fourth pathogen resistant to empiric therapy was Candida albicans.

^d^Concordant results in BC and NGS were observed in 3 samples collected after antibiotic administration.

^e^Bacterial resistance to the already administered antibiotic therapy was observed in 3 out of those 5 cases, of which 2 can be explained by intrinsic resistances of the detected bacteria.

Sensitivity and specificity of DISQVER® based on comparison to BC results were calculated for the 46 samples where results for both methods were available and were found to be 73.33% and 74.19%, overall accuracy was at 73.91% (Supplementary Table S3, Supplementary Fig. 1).

### Evaluation of NGS results

Of all 82 results in total, DISQVER® results were negative in 58 of 82 (70.7%) and positive in 24 of 82 (29.3%) cases. DISQVER® and blood cultures yielded concordant results in 9 of 15 cases (60%) in other words, the cfDNA of the same pathogen that was found in the blood culture was also found in mcfDNA sequencing (Table 3). DISQVER® results were deemed plausible in 75 of 82 cases (91.46%). In 4 of 7 implausible cases, DISQVER® results were deemed to be false negative. In these patients, blood cultures were positive for *K. pneumoniae*, *E. coli* and in two cases *S. epidermidis* associated with clinical signs of sepsis (Table 3).

In the three remaining inconclusive cases *Aspergillus niger* mfcDNA was identified in a sample from a patient without any clinical suspicion of aspergillosis. Additionally, mcfDNA of *Kytococcus sedentarius*, a bacterium unlikely to be the causative pathogen of sepsis, was detected, as well as mcfDNA of *Gardnerella vaginalis* in a term neonate who had received antibiotics prior to sample collection (Table 4).

**Table 4:** Matching clinical picture and routine microbiologic sampling to DISQVER® results in blood culture-negative cases.

Sample number	Group	Diagnosis	CRP (mg/l), IL-6 (pg/ml), Leukocytes (/µl)	Antibiotic prior to sampling	Empiric therapy –adequate?	Microbiology results, other than blood culture	Next-Generation sequencing result	Plausibility of NGS results
Concordance between NGS, microbiology (other than BC) and clinical course								
88	2	Chorioamnionitis, microcolon, small bowel perforation	248, 15844, 3050	No	Yes Van + Mero	Anal swab—*Enterobacter cloacae complex, Klebsiella Oxytoca*	*Enterococcus faecalis, Enterobacter hormaechei*	Yes
101	2	Gastric perforation, pulmonary hemorrhage, meconium plug syndrome, inguinal hernia	382, 1874, 2500	No	Yes Van + Pip/Taz	Pharyngeal swab— *Klebsiella oxytoca* Anal swab - *Klebsiella oxytoca, Klebsiella pneumoniae*	*Klebsiella pneumoniae, Lactococcus lactis, Citrobacter spp., Enterococcus faecalis*	Yes
122	2	Meconium ileus, necrotizing enterocolitis, small bowel perforation	20, 257, 16150	No	Yes Van + Pip/Taz	Pharyngeal swab— *Klebsiella oxytoca* Anal swab—*Klebsiella oxytoca*, *Citrobacter freundii*	*Klebsiella oxytoca, Klebsiella pneumoniae, Lactobacillus acidophilus, Salmonella enterica, Enterococcus faecium*	Yes
133	2	Sepsis, biventricular heart failure	15, 426836, 7800	No	Yes Van + Pip/Taz	Pharyngeal rinse— *Klebsiella oxytoca*	*Klebsiella pneumoniae, Klebsiella michiganensis, Klebsiella oxytoca*	Yes
Discordance between NGS, microbiology (other than BC) and clinical course								
14	2	Sepsis	67, 1367, 15040	No	Yes Amp/Sul	None	*Kytococcus sedentarius*	No
103	2	Left-sided congenital diaphragmatic hernia	28, 55, 18320	Yes	Yes Van + Pip/Taz	Skin and anal swab—Flora	*Gardnerella vaginalis*	No
111	3	Suspected late onset sepsis	<0.6, 3.8, 17100	No	No therapy	None	*Aspergillus niger*	No

NGS detected mcfDNA in seven cases while BCs remained negative. Antibiotics were administered to one of seven patients before sampling. Criteria for the plausibility of NGS results—concordance with microbiology and clinical course—were met by four of seven samples. Cases with mcfDNA of microorganisms not found in other microbiologic samples or unexplained by clinical course were deemed implausible (3/7). As NGS does not yield antibiotic susceptibilities, wild-type susceptibilities were assumed for evaluation of adequacy of empiric antibiotic therapy. Flora: this refers to bacteria commonly found at the target site. These are considered non-pathogenic if confined to that site, e.g., *Staphylococcus epidermidis* found in a skin swab. *Enterobacter cloacae* complex**:** Seven species of the genus *Enterobacter* belong to the *Enterobacter cloacae complex (ECLCO)*: *E. cloacae, E. asburiae, E. hormaechei, E. kobei, E. ludwigii, E. nimipressuralis, E. mori*. These species share phenotypic and, importantly, genotypic characteristics. MALDI-TOF MS often identifies *ECLCO* but may fail to reliably identify the different species that comprise the complex. Molecular methods like PCR or sequencing identify the genetic differences within the complex and can thus more reliably determine which species of ECLCO is present. For this reason, *Enterobacter cloacae* detected in blood culture is often reported as *ECLCO*, while NGS often reports the same bacteria to the exact species of *ECLCO*. This difference is of no clinical or therapeutic concern.

*Van* Vancomycin, Pip/Taz Piperacillin/Tazobactam, Amp/Sul Ampicillin/Sulbactam, *Mero* Meropenem, *CRP* C-reactive protein, *IL-6* interleukin 6, NGS next-generation sequencing, BC blood culture.

Positive percent agreement between DISQVER® results and the composite reference standard was calculated to be 82.61%, negative percent agreement 91.43% and overall accuracy were at 87.93% (Supplementary Table S4).

### Species identified in NGS results

**Fig. 2:**
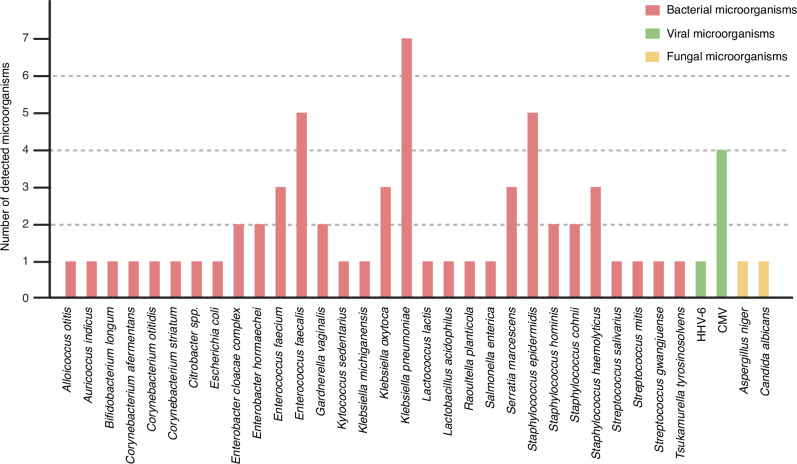
Microorganisms detected by DISQVER® mcfDNA sequencing. In the 24 positive DISQVER® results, 29 different bacterial microorganisms, along with two different viral and two different fungal microorganisms, were detected.

In the 24 positive DISQVER® results, 29 different bacterial pathogens, two different viral and two different fungal pathogens, were detected (Fig. 2). In 11 out of 24 (45.8%) of samples, DISQVER® detected mcfDNA of more than one different pathogen.

The sequencing results of 18 of 24 (75%) positive NGS samples yielded bacterial cfDNA of 29 different bacterial species or genera. In cases where identification down to the species levels of bacteria was not possible, the read counts for the genera or a group within the genera were reported (e.g., *Enterobacter cloacae complex* or *Citrobacter spp*.) (Fig. 2). DISQVER® detected mcfDNA of multiple bacteria in 11 of 24 samples (45.8%) while blood cultures showed the presence of more than 1 pathogen in only 1 of 15 positive blood cultures (6.7%). Generally, the presence cfDNA of multiple bacteria was associated with an abdominal focus of infection (6/11) and prematurity (10/11) while the detection of cfDNA of only one bacterial or fungal pathogen (9/24) was less associated with prematurity (2/9). See the supplementary for a breakdown of the NGS results, read counts and patient characteristics (Supplementary Table S1).

Viral cfDNA was detected in 4 of 24 DISQVER® positive samples (Human cytomegalovirus (CMV) in 3 samples, Human betaherpesvirus 6B (HHV-6B) in 1 sample). No fungal or bacterial cfDNA was detected in all 4 samples that showed the presence of viral cfDNA. Specific testing for viral pathogens during the clinical course was performed in 15 patients. These tests remained negative in 14 of 15 cases while 1 of 15 patients was CMV positive in tracheal secretions and urine. NGS also detected CMV cfDNA in this patients’ sample (Supplementary Table S2, patient 6). In the remaining 3 cases in which viral cfDNA was detected, there was no clinical suspicion of a viral infection and thus no virologic diagnostics were ordered by the clinicians.

Fungal cfDNA was detected in 2 of 24 DISQVER® positive samples - *Aspergillus niger* and *Candida albicans* mcfDNA, respectively. No bacterial or viral cfDNA was detected in the two samples with fungal cfDNA. The patient with Candida albicans cfDNA had matching blood culture results. This patients’ empiric antibiotic regimen did not include an antifungal drug. The NGS detection of *Aspergillus niger* cfDNA could not be confirmed by blood culture. Since there was no clinical suspicion of an aspergillosis, no further microbiologic tests were done on this patient.

### Potential value of positive NGS results

We recorded seven cases in which sequencing reported the mcfDNA of bacteria that could potentially have grown in blood cultures but that had negative blood culture results.

In three of seven cases, the microorganisms whose mcfDNA was detected by NGS were not found in routine microbiological screening and their presence was not explained by the patient’s clinical course (*Kytococcus sedentarius, Gardnerella vaginalis, Aspergillus niger*). Out of the seven samples, four showed high read counts of mcfDNA of bacteria that were also found in routine microbiological screening samples and matched their clinical diagnosis (Table 4).

### Impact of antibiotic therapy on pathogen detection

In 16 of the 58 infectious episodes (27.6%—Group 1, 2 and 3), patients were already on antibiotic treatment when blood cultures and NGS samples were collected (5 in group 1, 9 in group 2, and 2 in group 3, Supplementary Table S2). Blood cultures did not exhibit growth, nor could mcfDNA be detected, in 11 of those 16 pre-treated cases (only group 2 and 3). Bacteria grew in the blood cultures of 5 of 16 patients (all group 1) despite prior antibiotic therapy (*Klebsiella pneumoniae, Serratia marcescens, Escherichia coli*, Acinetobacter ursingii *and Staphylococcus epidermidis*). Cultures from three of these five patients grew bacteria resistant to the empiric therapy chosen, of which two were due to an expected resistant phenotype. DISQVER® results were congruent in three of five cases (Table 3). McfDNA was detected in 5 of the 16 pretreated samples (31.25%) (*Klebsiella pneumoniae, Serratia marcescens, Escherichia coli*, CMV *and Gardnerella vaginalis*).

In 42 of 58 infectious episodes (72.4%) no antibiotics had been administered prior to sampling. The detection rate here was 23.8% (10/42) for blood cultures while NGS detection rate was 38.1% (16/42).

## Discussion

In recent years, the collection of cell-free DNA from patient samples has found increasing application in clinical medicine, personalized medicine, tumor diagnostics, prenatal diagnostics or pathogen identification.^20,24–27^ Sequencing of mcfDNA may increase detection of fastidious bacteria such as anaerobes or intracellular organisms.^20,28^ Standard pediatric blood culture bottles used in our clinic and this study (BD BACTEC™ Peds Plus™) contain an aerobic environment, since most bacterial infections are caused by aerobic or facultative anaerobic bacteria. This decreases the chance of culturing strictly anaerobic bacteria commonly seen in intraabdominal infections.^29,30^ Due to competition among various bacteria in the shared culture medium blood cultures often identify only a single bacterial species (Supplementary Table S1).^31^ NGS reported mcfDNA of multiple pathogens, especially in premature patients or patients with intraabdominal infections. Whether this represents a true polymicrobial infection, common in patients with intraabdominal infections,^32^ or transient bacteremia caused by dysfunctional or immature mucosal barriers, remains unclear.

NGS has the potential to increase sensitivity in diagnosis of sepsis or BSIs, since it is applicable even after initiation of an antibiotic regimen, whereas culture may yield limited results in this setting.^20,33^ Contrarily, we did not observe a higher rate of pathogen detection using sequencing compared to blood cultures in patients who received antibiotics prior to sampling in this study. Previous studies described a relatively short half-life of human cfDNA of 16 min to 2 h.^34,35^ The short half-life of cfDNA or the absence of a metastatic focus of infection with continuous release of mcfDNA might explain why we did not observe an increased detection rate using NGS compared to culture in patients treated with antibiotics prior to sample collection.^36^

The challenge associated with this emerging technology lies in comprehending whether the identified pathogen is either of acute clinical relevance, or the result of a contamination of reagents/laboratory processes, or reflects a transient bacteremia that arises from an impaired function of mucosal barriers.^26^ The detection of *Aspergillus niger* cfDNA in NGS, without any clinical signs of aspergillosis, is likely indicative of contamination. To decrease the risk of plasma sample contamination in future studies, we recommend the use of sampling vials containing stabilization media which inactivates pathogens but preserves nucleic acid for NGS. Additionally, it’s crucial to assess whether mcfDNA represents active infection or merely remnants of pathogens previously cleared by immune cells in situations of suspected impaired mucosal barrier function, as it is often seen in this patient population.^20^ This is an important issue which still needs to be further investigated when using NGS for sepsis diagnosis in preterm and term infants. To prevent overtreating potential flora or contaminants detected by NGS, we recommend seeking guidance from an infectious disease specialist or microbiologist as well as considering the clinical context and other microbiologic findings when making treatment decisions.

DISQVER® has the potential to detect mcfDNA of bacteria that have been traditionally classified as part of the normal flora, rarely pathogenic or fastidious bacteria missed by conventional cultural diagnostic techniques. At present it is unclear how relevant the detection of mcfDNA from such bacteria is for clinicians and patients. By way of example, we may refer to the detection of *Corynebacterium otitidis, Alloiococcus otitidis, Raoultella planticola* and *Corynebacterium afermentans* mcfDNA in the DISQVER® samples, while cultures grew *E. cloacae* complex, *S. epidermidis* and *S. capitis*. In the setting of sepsis diagnosis the NGS results were deemed implausible since it likely represents detection of flora rather than pathogenic bacteria (Patient 89, Table 3).

Currently, one of the main limitations of DISQVER® is its inability to provide information regarding antimicrobial susceptibility. In the future, it may be possible not only to identify the pathogen, but also to detect resistance genes or even patterns of specific mcfDNA that can be associated with certain patterns of previously described antimicrobial resistance. DNA fragments containing genes that confer resistance to certain antibiotics can be detected by sequencing.^37^ However, it is known that genotypic resistance to specific antibiotics may not always be expressed phenotypically (silent mutation) and phenotypical resistance is not easily predictable from the genotype, since it is infrequently linked to single genes.^38–40^ Future studies are needed to determine whether sequencing mcfDNA can predict antibiotic resistance either by detection of resistance genes or by genotype-phenotype matching, and how this will impact clinical decision-making.

The identification of a pathogen alone, even without susceptibility results, may also have significant clinical implications such as modification or escalation of treatment. A good example is the identification of a fungal pathogen, like *Candida albicans*, since empiric antibiotic treatments usually do not contain an antifungal drug (patient 57, Table 3 or Supplementary Table S1). Another example from this study is an infection with *Serratia marcescens* in a preterm infant initially treated with ampicillin/sulbactam and tobramycin (patient 116, Table 3 or Supplementary Table S1). The typical empiric regimen for neonatal sepsis comprises ampicillin/sulbactam combined with an aminoglycoside ^41^ (tobramycin in our setting). However, current EUCAST recommendations advise against aminoglycoside monotherapy for severe infections like sepsis.^42,43^ Given *Serratia marcescens’* intrinsic resistance to ampicillin/sulbactam,^44^ following the empiric regimen would essentially result in monotherapy. Furthermore, there is a growing trend of aminoglycoside resistance among *Serratia marcescens* strains.^45,46^ It is important to note that since this was primarily a feasibility study, the NGS results were not reported back to the clinicians in real-time and thus did not influence clinical decision-making. In a retrospective analysis of the empiric antibiotic therapy, clinical picture, blood culture, screening and NGS results by infectious disease experts and microbiologists this NGS result would have led to a change in therapy.

The present study revealed seven cases in which a positive sequencing result was obtained, and blood cultures were negative. Four of seven individuals presented considerable read counts of mcfDNA from the same bacteria identified in routine microbiological sampling and that was consistent with their clinical diagnosis (Table 4), suggesting that NGS may have identified infections that were missed by culture. In retrospect, the empiric antibiotic therapies subsequently initiated in these patients were adequate in 6 out of 7 cases thus NGS results would have not led to a change in therapy. The remaining case, where therapy might have been inadequate, was deemed a contaminant (*Aspergillus niger*).

Additionally, we recorded four cases in which a positive blood culture was reported but NGS was negative. Among these four cases, two involved the growth of *Klebsiella pneumoniae* and *Escherichia coli* in culture, while NGS results were negative. In the remaining two cases *Staphylococcus epidermidis* was cultured in a patient with a suspected CRBSI but NGS did not generate reads for this bacterium. Considering the possibility of CRBSI, the time to positivity (TTP) of the two BCs was evaluated. The staphylococci in both cultures exhibited growth within 17 and 37 h, respectively. The clinical suspicion of a CRBSI and this relatively short TTP may hint at a true bacteremia rather than contamination.^47^ (Sample 3 and 4, Supplementary Table S2). The DISQVER® results of these four cases were considered unlikely.

In our study, we have discovered an additional potential application for NGS, namely, the identification of viral pathogens (DNA viruses), such as CMV. It was not the focus of our study whether DISQVER® could serve as an appropriate diagnostic tool for detecting infections caused by DNA viruses but this topic warrants further investigation in future research.

Some limitations in the design of this study need to be acknowledged. The heterogeneity of previously published LOS definitions^23^ and the relatively unspecific clinical symptoms associated with bloodstream infections and clinical sepsis might be a limitation for the group allocation in our study. It is noteworthy that 8 of 26 patients in group 2, who exhibited clinical signs of sepsis and elevated inflammatory markers, received antibiotic therapy for only 5 days. These patients demonstrated clinical improvement and survived, suggesting that their initial sepsis diagnosis may have been less likely. In order to compare the diagnostic value of blood cultures and NGS, the validation group patients should have undergone blood culture collection. However, this was not feasible due to ethical considerations. Consequently, we lack information about potential blood culture contaminations in the validation group while the mcfDNA levels in most patients in this group were too low for detection by DISQVER®. In adults DISQVER® has the potential to distinguish between minimal amounts of mcfDNA and levels of mcfDNA that are clinically significant and indicative of infection.^17^ Additional data is needed to determine whether the DISQVER® algorithm needs to be further adapted to lower neonatal mcfDNA thresholds, sequencing libraries updated or if a higher amount of plasma is required to reduce the high number of samples that were not processable by NGS (52/134) and reduce the detection or reporting of flora. The lack in sufficient cfDNA in the sample material, especially in the validation group, might be attributed to usually lower cfDNA concentrations in healthy individuals in comparison to septic patients, as already observed for adult patients but recently also shown for neonates.^48^ Additionally, due to the limited sample material it was not possible to compare to duplicates/pairs for blood culture and for NGS or even to a third diagnostic technique, such as PCR, which would have been especially useful in case of discordant results. Furthermore, the study population was heterogeneous, including term and preterm neonates, and neonates with congenital anomalies such as congenital diaphragmatic hernia and congenital heart defects, as well as patients who did or did not undergo surgical therapy.

## Conclusion

Our findings demonstrate that DISQVER® can detect mcfDNA from both pathogenic and likely non-pathogenic bacteria, fungi and viruses, even when blood cultures yield negative results. While NGS has the potential to refine conventional diagnostic methods, such as in intraabdominal polymicrobial infections, and may lead to improved, pathogen-directed treatment, our study also revealed false negative NGS results. Notably, we found no improved detection rates with NGS compared to blood culture in patients who had antibiotics prior to sample collection. In summary, interpreting NGS results remains challenging, underscoring the necessity for consultation with infectious disease specialists and integration with clinical and microbiological data to ensure optimal clinical utility.

## Supplementary Information

Supplementary Material

Supplementary tableS1

Supplementary tableS2

## Data Availability

The datasets generated and analyzed during the current study are not publicly available. Most data are available in the supplementary tables. Further information is available from the corresponding author upon reasonable request.
