# Supplementary material for: Microbial cell-free DNA-sequencing as an addition to conventional diagnostics in neonatal sepsis
Source: Pediatr Res. 2024 Aug 14;97(2):614–24. doi: 10.1038/s41390-024-03448-1 (PMC12015174; doi:10.1038/s41390-024-03448-1)
Supplement: Supplementary file 1 — Supplementary Material [file 41390_2024_3448_MOESM1_ESM.pdf]

Supplementary Material: Microbial cell-free DNA-sequencing as an addition to conventional diagnostics in neonatal sepsis.

Julian Balks [1,2], Silke Grumaz [3], Sonia Mazzitelli [3], Ulrike Neder [3], Lotte Lemloh [1], Tamene Melaku [1], Kirsten Glaser [4], Andreas Mueller [1], Florian Kipfmueller [1].

Affiliations

[1] Department of Neonatology and Pediatric Intensive Care, Children's Hospital, University Hospital Bonn, Bonn, Germany

[2] Institute of Medical Microbiology, Immunology and Parasitology (IMMIP), University Hospital Bonn, Bonn, Germany

[3] Noscendo, Duisburg, Germany

[4] Department of Neonatology, University Hospital Leipzig

Corresponding Author

Florian Kipfmueller MD

Department of Neonatology and Pediatric Intensive Care,

Children's Hospital, University of Bonn,

Venusberg-Campus 1, 53127 Bonn, Germany

Phone: +49 – 228 – 287 37843

E-mail: florian.kipfmueller@ukbonn.de

Supplementary Table S1 - Positive NGS results

Supplementary Table S2 – Cohort overview

Supplementary Table S3 Sensitivity and Specificity based on 46 samples with results for both tests

		Blood culture	
		+	-
DISQVER	+	11	8
	-	4	23

Statistic	Value	95% confidence interval (CI)
Sensitivity	73.33%	44.90% to 92.21%
Specificity	74.19%	55.39% to 88.14%
Positive Likelihood Ratio	2.84	1.45 to 5.56
Negative Likelihood Ratio	0.36	0.15 to 0.85
Disease prevalence	32.61%	19.53% to 48.02%
Positive Predictive Value	57.89%	41.29% to 72.89%
Negative Predictive Value	85.19%	70.78% to 93.17%
Accuracy	73.91%	58.87% to 85.73%

n=11, samples that were NGS positive and BC positive (this includes 2 samples where NGS and BC differed); n=4, samples with positive BC and negative NGS; n=8, samples that were NGS positive and BC negative; n=23, samples that were blood culture and NGS negative. **Abbreviations**: Next-Generation sequencing (NGS), blood culture (BC)

Supplementary Table S4 Positive percent agreement and negative percent agreement based on composite reference standard for 58 samples (Group 1, 2 and 3).

		Composite STD (standard deviation)	
		+	-
DISQVER	+	19	3
	-	4	32

DISQVER results:

true positive (n=19)

false positive (n=3)

false negative (n=4)

true negative (n=32)

Statistic	Value	95% confidence interval (CI)
Positive Percent Agreement	82.61%	61.22% to 95.05%
Negative Percent Agreement	91.43%	76.94% to 98.20%
Positive Likelihood Ratio	9.64	3.21 to 28.90
Negative Likelihood Ratio	0.19	0.08 to 0.47
Disease prevalence	39.66%	27.05% to 53.36%
Positive Predictive Value	86.36%	67.87% to 95.00%
Negative Predictive Value	88.89%	76.55% to 95.15%
Accuracy	87.93%	76.70% to 95.01%

True positive (n=19), samples that were NGS positive and plausible; False positive NGS results (n=3), sample number 14 (*Kytococcus sedentarius*), 103 (*Gardnerella vaginalis*) and 111 (*Aspergillus niger*); False negative (n=4), samples that were BC positive and NGS negative; True negative (n=32), samples that were blood culture and NGS negative. **Abbreviations**: Next-Generation sequencing (NGS), blood culture (BC)

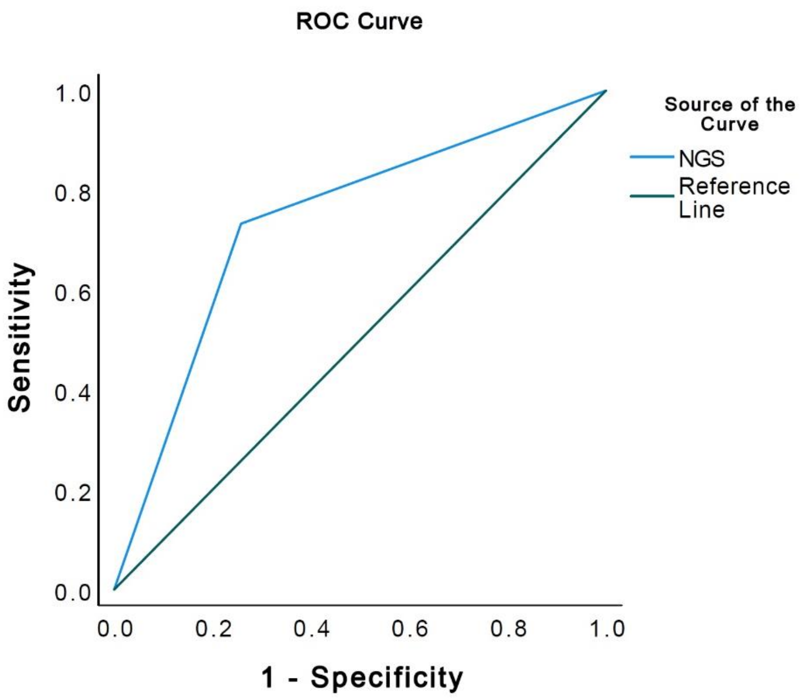

Supplementary Figure 1 - Receiver operating curve for NGS to predict a positive BC. The area under the curve was 0.738 (95% CI: 0.579 -0.896), p=0.003.
